# Computer-assisted quantification of motile and invasive capabilities of cancer cells

**DOI:** 10.1038/srep15338

**Published:** 2015-10-21

**Authors:** Karthiga Santhana Kumar, Max Pillong, Jens Kunze, Isabel Burghardt, Michael Weller, Michael A. Grotzer, Gisbert Schneider, Martin Baumgartner

**Affiliations:** 1 Department of Oncology, Children’s Research Center, University Children’s Hospital Zürich, August-Forel Strasse 1, CH-8008 Zürich, Switzerland; 2 Department of Chemistry and Applied Biosciences, ETH Zürich, Vladimir-Prelog-Weg 4, CH-8093 Zürich, Switzerland; 3 Department of Neurology, University Hospital Zürich and University of Zürich, Frauenklinikstrasse 26, CH-8091 Zürich, Switzerland; 4 Department of Oncology, University Children’s Hospital Zürich, Steinwiesstrasse 75, CH-8032 Zürich, Switzerland

## Abstract

High-throughput analysis of cancer cell dissemination and its control by extrinsic and intrinsic cellular factors is hampered by the lack of adequate and efficient analytical tools for quantifying cell motility. Oncology research would greatly benefit from such a methodology that allows to rapidly determine the motile behaviour of cancer cells under different environmental conditions, including inside three-dimensional matrices. We combined automated microscopy imaging of two- and three-dimensional cell cultures with computational image analysis into a single assay platform for studying cell dissemination in high-throughput. We have validated this new approach for medulloblastoma, a metastatic paediatric brain tumour, in combination with the activation of growth factor signalling pathways with established pro-migratory functions. The platform enabled the detection of primary tumour and patient-derived xenograft cell sensitivity to growth factor-dependent motility and dissemination and identified tumour subgroup-specific responses to selected growth factors of excellent diagnostic value.

Cell migration is fundamental for numerous cellular physiological processes and the de-regulation of its homeostatic control is causative for human diseases ranging from autoimmunity and inflammation to cancer metastasis^1^,^2^,^3^. Cell migration is controlled by the integration of mechanical and chemical cues and their impact on the executing machinery, the cellular cytoskeleton^4^,^5^,^6^, which defines cellular morphology and morphodynamics by a broad range of processes^7^,^8^,^9^. Hence, aberrant induction and maintenance of a migratory phenotype could be caused by a plethora of molecular processes coupled to cellular morphodynamics. Deeper insights into these processes and the systematic study of the underlying mechanisms require innovative, high-throughput tools that enable multidimensional visualization and quantification of cell motile behaviour in space and time.

According to the World Cancer Report 2014 of the World Health Organization metastatic dissemination of tumour cells is the leading cause of death in cancer patients, and understanding of the causative events of cancer metastasis will be essential for developing effective targeting strategies^3^,^10^. The identification of the relevant cellular processes remains a formidable challenge because of the large number of potential targets to be explored and the difficulties to reproducibly track altered cell motility. Cell migration is a graded process, with small alterations caused by subtle changes in the cellular motility machinery. Many cell-based assays have been developed to monitor the behaviour of cells on two-dimensional (2D) surfaces or inside three-dimensional (3D) matrices^11^,^12^,^13^,^14^,^15^. Several assays tackled high-throughput quantification of cell motility in 2D^15^,^16^,^17^. Assays to automatically determine the dissemination range of cells migrating in 3D are not yet available, mostly because of the difficulties to efficiently measure the distance between origin and endpoint of migration of cells migrating detached from a solid substrate. To enable cell motility quantification in 2D and 3D, we have assembled a package of three cell migration assays and combined them with automated imaging and computational image analysis. This new approach now allows the efficient evaluation of migration-regulating functions of chemical and mechanical cues over a wide range of conditions.

Medulloblastoma (MB) is a highly malignant embryonal neuroepithelial tumour of the cerebellum with a tendency to metastasize within the central nervous system^18^. Genomic analyses classified MB into the four molecular subgroups wingless (WNT), sonic hedgehog (SHH), group 3 and group 4^18^,^19^. Macroscopic and microscopic evidence of metastases is considered a high risk factor and despite aggressive treatment regimens, one-third of patients succumb to the disease^18^. Metastatic dissemination is specifically associated with tumours of the MB subgroups 3 and 4^18^. However, it can also be triggered in the SHH subgroup by the ectopic expression of selected putative driver genes such as Eras, Lhx1, Ccrk, and Akt^20^ or by the activation of growth factor signalling pathways such as hepatocyte growth factor (HGF)-c-Met signalling^21^. The mechanisms triggering and maintaining MB dissemination are largely unknown. We hypothesised that growth factors trigger detachment and dissemination of cells from the primary tumour. Therefore, we tested the migratory response of established SHH MB lines^22^, medulloblastoma patient-derived xenograft (Med PDX) and primary MB lines to growth factor stimulation and thereby explored the potential impact of such factors to metastatic dissemination. We show the validation of our automated cell motility analysis platform and demonstrate its efficacy and functionality to determine factors driving the dissemination of both established cancer cell lines and primary tumour cells.

## Results

### Automated quantification of cell dissemination

**Figure 1:**
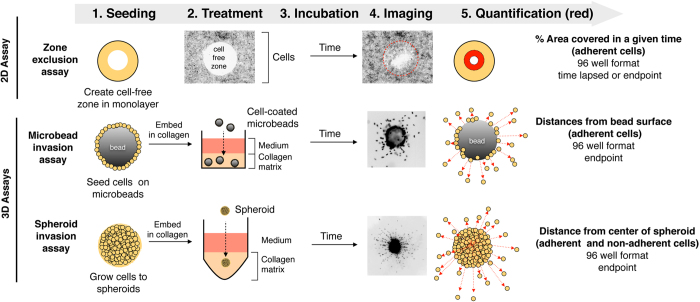
Schematic overview of 2D and 3D cell-based dissemination quantification assays. Cells are depicted as light-brown, filled circles. Alterations in cell localization at a given time point are highlight in red.

To explore extrinsic and cell intrinsic factors controlling collective cancer cell dissemination in 2D, we used the zone exclusion assay^17^, which provides circular cell free surfaces of identical area and allows the quantification of area covered by cells over time (Fig. 1). A high-throughput assay was recently developed to quantify the number of cells disseminated from spheroids into collagen I matrix^14^. However, this assay does not allow the quantification of the cell spreading distances. Moreover, the mode of cell migration is semi-3D as the cells are allowed to attach to and migrate on a solid support. To overcome these limitations and to quantify cell dissemination and invasion in 3D, we established the microbeads cell dissemination/invasion and the spheroid cell dissemination/invasion assays. Both assays are true 3D assays that allow the cells to disseminate fully detached from a defined reference point into a 3D matrix. The reference points are the surface of the microbeads and the centres of the spheroid, respectively (Fig. 1). The main difference between the microbeads and the spheroid invasion assay is that former measures dissemination/spreading of cells grown into a two-dimensional monolayer on the bead surface into the collagen matrix (2D to 3D), whereas latter measures dissemination/spreading of cells from three-dimensional cell aggregates (3D to 3D).

**Figure 2:**
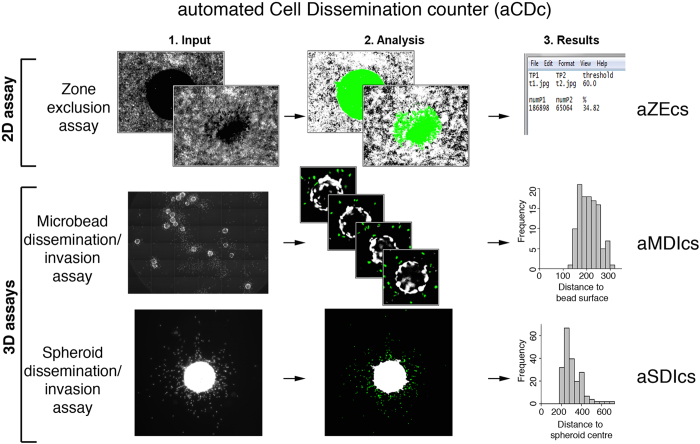
Schematic overview of working principle of 2D and 3D cell dissemination and invasion counter software tools: aZEcs (automated Zone Exclusion counter software), aMDIcs (automated Microbeads Dissemination/Invasion counter software) and aSDIcs (automated Spheroid Dissemination/Invasion counter software). Green area in aZECs represents area recognized and quantified by software and green nuclei highlight the cells recognized and counted by aMDIcs and aSDIcs.

The zone exclusion assay works best with cells displaying cytosolic fluorescence (e.g. EGFP, lifeact-EGFP, or fluorescent live cell stains), whereas the 3D assays require nuclear staining for better separation of individual cells. For each assay we developed the corresponding software tools (Fig. 2), which either progressively quantify the area covered by cells (2D zone exclusion) or number and distance of disseminated cells (3D assays). We named the software according to their working principles: aZEcs (automated Zone Exclusion counter software), aMDIcs (automated Microbead Dissemination/Invasion counter software) and aSDIcs (automated Spheroid Dissemination/Invasion counter software). A program suite referred to as automated Cell Dissemination counter (aCDc) consisting of three open source, executable Java programs (.jar) files can be downloaded through the following web link http://www.infozentrum.ethz.ch/uploads/user_upload/Software/.

### Assay Validation

**Figure 3:**
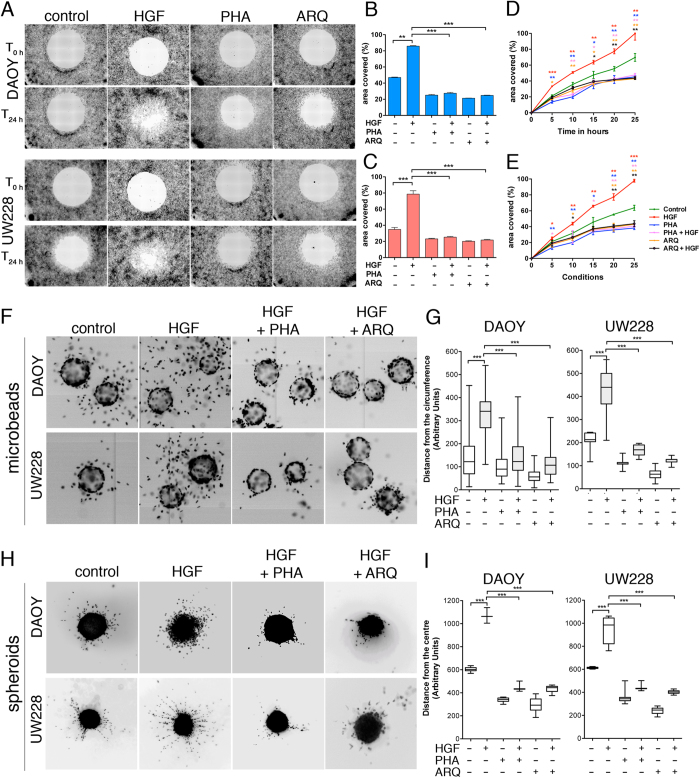
aZEcs, aMDIcs and aSDIcs quantifications reliably confirm manual measurements of HGF-induced cell dissemination in the presence of 10% serum. (**A**) Representative images of 100× magnified zones of exclusion in monolayers of LA-EGFP expressing DAOY or UW228 cells in 96 well plate. Images are inverted greyscale of LA-EGFP fluorescence at 0 (T_0h_) and 24 h (T_24h_) −/+ stimulation with 20 ng/ml HGF and/or treatment with c-Met inhibitors PHA665752 and ARQ197 (125 nM each). (**B,C**) Means and SDs of % area covered from three independent zone infiltration experiments using aZEcs in DAOY (**B**) or UW228 (**C**) at T_24h_ after HGF stimulation and/or inhibitor treatment. (**D**,**E)** Time-lapsed mean and SD quantifications of % area covered from three independent zone infiltration experiments using aZEcs in DAOY (**D**) or UW228 (**E**) after HGF stimulation and/or inhibitor treatment. (**F)** Representative images of 100x magnified microbeads coated with DAOY or UW228 cells after 24 h −/+ stimulation with 20 ng/ml HGF and/or treatment with c-Met inhibitors PHA665752 and ARQ197 (125 nM each). Inverted greyscale images of Hoechst-stained nuclei are shown. (**G)** Quantification of distance from the surfaces of the microbeads using aMDIcs. Box plots with pooled data of two independent experiments and whiskers min to max are shown. (**H)** Spheroid invasion assay with DAOY and UW228 cells. Treatment and imaging as in F), except that 50× magnification was used. (**I)** Quantification of distance from centres of spheroids using aSDIcs. Box plots with pooled data of two independent experiments and whiskers min to max are shown.

HGF/c-Met signalling promotes motility of MB tumour cells, and is associated with an aggressive invasive phenotype in patients^21^,^23^. Therefore, we used HGF stimulation to validate our assays in combination with aCDc. We treated the human SHH MB cell lines DAOY and UW228 in the zone exclusion assay with 20 ng/ml HGF in the absence or presence of pharmacological c-Met inhibitors (PHA665752 or ARQ197, 125 nM). Using aZEcs, we analysed images acquired under both 10% serum (Fig. 3A–E) and serum-free conditions (Supplementary Fig. S1). Both cell lines stably express lifeact-EGFP (LA-EGFP) for monitoring F-actin dynamics. Consistent with our previous quantifications^23^, blocking c-Met with pharmacological inhibitors reduced basal migration and prevented HGF-induced motility (Fig. 3B,C and Supplementary Fig. S1A, B). An inherent problem of the zone exclusion assays is that cell proliferation could falsify motility outputs based on area covered. We therefore used time-lapse video microscopy imaging, which provides refined information on cell dissemination at time points (5–10 h) when proliferation effects can be considered marginal. Indeed, we found that HGF treatment accelerated cell migration significantly (T-test, *p *= 0.00014) already within 5 h incubation under 10% serum (Fig. 3D,E) and serum-free conditions (T-test *p *= 0.0276, Supplementary Fig. S1C, D). Consistently, this pro-migratory effect of HGF was blocked with pharmacological c-Met inhibitors, which caused the reduction of cell migration by 50% within 5 h incubation in DAOY (Fig. 3D, Supplementary Fig. 1C) and UW228 cells (Fig. 3E, Supplementary Fig. 1D).

We used the same cell lines to assess the effects of HGF, PHA665752 and ARQ197 on cell dissemination in the 3D environment. The images were acquired either on a Molecular Device automated microscope (microbeads) or on a Zeiss AxioObserver (spheroids) and quantified using aMDIcs and aSDIcs. We found that treatment with HGF induced extensive invasiveness into collagen, both from microbeads (Fig. 3F,G, Supplementary Fig. S2, S3E Supplementary videos 1,2,3,4) and spheroids (Fig. 3H,I, Supplementary Fig. S3F), which was abolished with concomitant PHA665752 or ARQ197 treatments.

Overall, we show that the motility assays developed, in combination with aCDc, provide versatile end-point or time-lapse quantification means that can rapidly and accurately measure cell migration and invasiveness.

### Differential impact of growth factors and cytokines on MB cell migration and invasiveness

**Figure 4:**
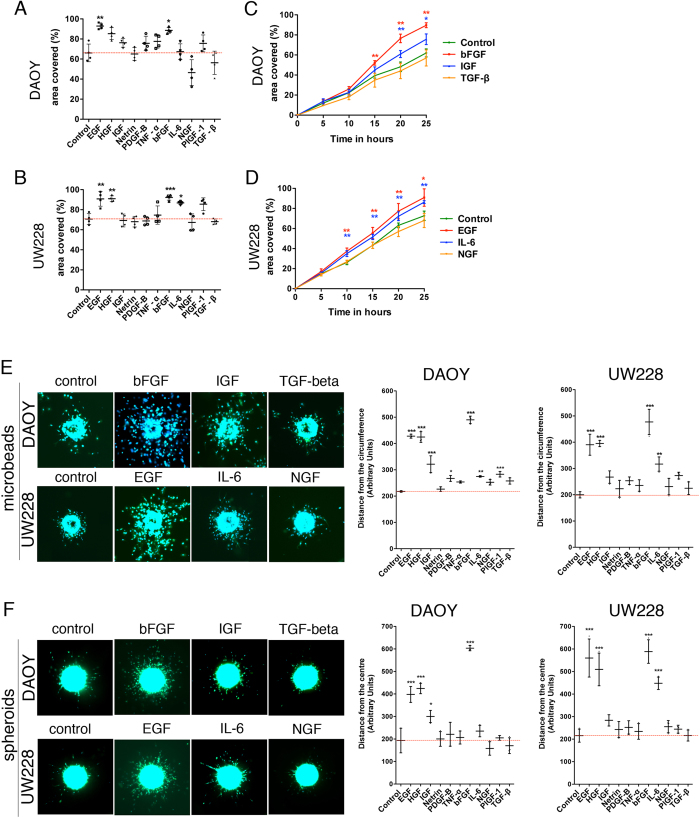
Selective induction of cell dissemination with growth factors in the presence of 10% FCS. (**A,B**) End-point quantification of % area covered in zone infiltration assay using aZEcs. Means and SDs of three independent experiments with DAOY (**A**) or UW228 (**B**) cells at T_24h_ after stimulation with factors as indicated are shown. Concentrations of growth factors/cytokines were as follows: HGF (20 ng/ml), EGF 30 ng/ml), IGF (20 ng/ml), netrin (200 ng/ml), PDGF-B (20 ng/ml), TNF-α (25 ng/ml), bFGF (100 ng/ml), IL-6 (20 ng/ml), NGF (50 ng/ml), TGF-β (20 ng/ml) and PlGF-1 (10 ng/ml) (**C,D)** Time-lapsed mean and SD quantifications of % area covered from three independent zone infiltration experiments using aZEcs in DAOY (**C**) or UW228 (**D**) cells stimulated with factors as indicated. (**E)** Representative images of 100× magnified microbeads coated with DAOY or UW228 cells after 24 h −/+ stimulation with bFGF, IGF or TGF-beta (DAOY) or EGF, IL-6 or TGF-beta (UW228). LA-EGFP in green, Hoechst staining in blue. Right panels: quantification of means and SDs of cell dissemination/invasion from three independent experiments using aMDIcs. Concentrations of growth factors/cytokines as in 4(**A,B**). **(F**) Representative images of 50× magnified spheroids of DAOY or UW228 cells after 24 h −/+ stimulation with bFGF, IGF or TGF-beta (DAOY) or EGF, IL-6 or NGF (UW228); LA-EGFP in green, Hoechst staining in blue. Right panels: Quantification of distance of cell dissemination/invasion using aSDIcs. Concentrations of growth factors/cytokines as in 4(**A,B**).

Growth factors in the tumour microenvironment play a critical role in tumour initiation, progression and metastasis^24^. Therefore, we determined the effect of predominant growth factors/cytokines present in the tumour microenvironment of MB on cell migration and invasiveness. We analysed the effect of HGF (20 ng/ml), EGF (30 ng/ml), IGF (20 ng/ml), netrin (200 ng/ml), PDGF-B (20 ng/ml), TNF-α (25 ng/ml), bFGF (100 ng/ml), IL-6 (20 ng/ml), NGF (50 ng/ml), TGF-β (20 ng/ml) and PlGF-1 (10 ng/ml) using the 2D zone exclusion assay and quantified it with aZECs. HGF, EGF and bFGF significantly promoted cell migration in DAOY and UW228 cells under both 10% serum and serum free conditions (One-way ANOVA *p *≤ 0.05). IL-6, IGF and PlGF-1 moderately influenced cell migration while the other factors did not significantly impact on cell migration (Fig. 4A,B and Supplementary Fig. S3A, B). Based on the above results, we were able to classify the growth factors and cytokines as strong, moderate and weak promoters of cell migration. Using time-lapse video microscopy, we found that bFGF promoted cell migration significantly within 15 h in DAOY cells (T-test, *p *= 0.0026), while IGF’s effect was significant (T-test *p *= 0.0197) only after 20 h (Fig. 4C and Supplementary Figure S3C). EGF and IL-6 significantly increased (T-test, *p *= 0.00741 and 0.0136, respectively) UW228 cell migration after 10 h of incubation compared to untreated control (Fig. 4D and Supplementary Fig. S3D).

To better understand the relationship between tumour microenvironment parameters and cell invasion, we studied the effects of HGF, EGF, IGF, netrin, PDGF-B, TNF-α, bFGF, IL-6, NGF, TGF-β and PlGF-1 in the 3D environment. We found that the signature pattern of promoting cell migration in 2D environment as strong, moderate and weak was conserved in the 3D environment. HGF, EGF and bFGF induced cell invasiveness in both microbead invasion and spheroid invasion assay under 10% FCS (Fig. 4E,F) and serum-free conditions (Supplementary Fig. S3E, F). Precise quantification using aMDIcs and aSDIcs showed that IGF had a moderate influence on cell invasion of DAOY but not of UW228 cells (Fig. 4E,F, Supplementary Fig. S3E, F). Similarly, IL-6 had a moderate influence on cell invasion of UW228 but not of DAOY cells (Fig. 4E,F, Fig. Supplementary Fig. S3E, F), depicting the differential behaviour of the two cell lines with similar phenotypes.

Taken together, we showed that there are strong, moderate and weak promoters of cell dissemination and invasion in the 2D and 3D environment and that each cell type responds differently to these stimuli. The distinct reactions of each cell type to different stimuli can be accurately identified and quantified using aCDc.

### Evaluation of sub group-specific sensitivities to promigratory stimuli

**Figure 5:**
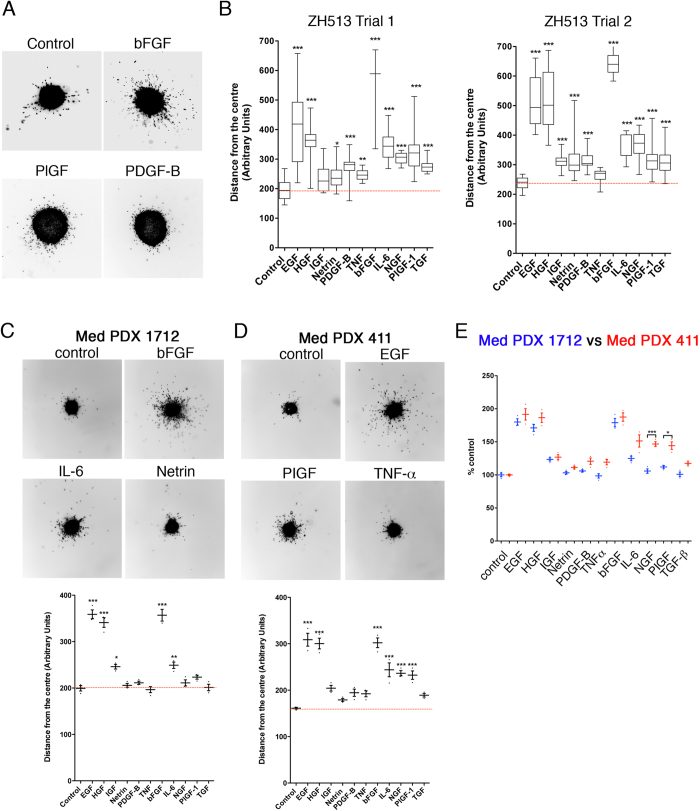
Quantification of growth factor-induced dissemination/invasion of primary MB tumour cells. (**A)** Representative images of 50× magnified spheroids of primary MB tumour cells after 24 h −/+ stimulation with bFGF, PIGF and/or PDGF-beta. Inverted greyscale images of Hoechst-stained nuclei are shown. (**B)** Quantification of distance from centres of spheroids in two experimental replicas using aSDIcs. Concentrations of growth factors/cytokines as in 4(**A**,**B**). Box plots with whiskers min to max are shown. Representative images of 50× magnified spheroids of Med PDX 1712 (**C)** and Med PDX 411 (**D)** and the quantification of average cell disseminations using aSDIcs. Concentrations of growth factors/cytokines as in 4A,B. Mean and S.D. of three experiments are shown. (**E)** Dot plot compares average dissemination of Med PDX 1712 (blue) and Med PDX 411 (red) relative to unstimulated controls.

Differences in sensitivity to growth factor-induced cell motility between the closely related DAOY and UW228 lines implied that cells of individual tumours might respond differently to growth factor stimuli. We therefore tested whether patients could be stratified based on growth factor sensitivity, ultimately for adapting therapy schemes to growth factor sensitivities of the tumor. Therefore, we analysed the effects of the various growth factors/cytokines on the primary line ZH-513, which we established from a non-SHH/WNT MB tumour sample (Fig. 5A). Of note, HGF, EGF and bFGF had the maximum influence on cell invasiveness (Fig. 5B), similar to that observed in DAOY and UW228. However, we observed significant differences (One-way ANOVA *p *≤ 0.0001) in the cell motility of ZH-513 cells when stimulated with NGF, PlGF-1 and TGF-β compared to DAOY (Fig. 4F) or Med PDX 1712 cells (Fig. 5C), indicating a difference in sensitivity and/or response among MB sub-groups. To check whether the same signature of growth factor-induced cell invasion is maintained in the ZH-513 cells, we repeated the same experiment after 10 days of culturing the primary cells *in vitro* (Fig. 5B, trial 2). The ZH-513 cells maintained their signature pattern of cell invasiveness with HGF, EGF and bFGF being strong promoters of invasiveness and IL-6, NGF, PlGF-1 and TGF-β being intermediate ones (Fig. 5B). These results demonstrate that primary patient material can be grown *in vitro* and tested using the spheroid invasion assay and aSDIcs for efficient diagnosis.

To further confirm the usability of aSDIcs for diagnostic evaluation of cell dissemination, we studied the impact of growth factors and cytokines on the MB Med PDX cell lines Med 1712 and Med 411. Invariably, HGF, EGF and bFGF had a strong influence on cell dissemination in both PDX lines (Fig. 5C,D). Differences between the two lines further substantiate the fact that response to growth factors can be used to identify aberrantly activated signalling pathways. IGF and IL-6 significantly increased (One-way ANOVA, *p *≤ 0.05 and 0.01, respectively) cell dissemination in Med 1712, (Fig. 5C), while IL-6, NGF and PlGF-1 did so in Med 411 (One-way ANOVA, *p *≤ 0.05, Fig. 5D) compared to untreated controls. Direct comparison of the relative impact of all factors between Med 1712 and Med 411 also revealed significant differences when the cells were stimulated with either NGF or PlGF-1 (One-way ANOVA, *p *≤ 0.0001 and 0.05, respectively, Fig. 5E). The results above indicate that whether or not the cells respond to NGF and PlGF-1 may be used as markers to differentiate the two PDX cell lines and likely also other SHH and group 3-derived MB tumour cells.

Collectively, we have revealed similar and differential growth factor responses of primary patient material and PDX cell lines compared to the established MB cell lines. These different migratory responses of the samples to growth factor stimulation can be easily detected and quantified using aSDIcs and may be exploited to sub-group patient samples and PDX cell lines based on their sensitivities to growth factor stimuli.

## Discussion

We have developed an assay platform to quantify cancer cell dissemination in 2D and 3D environments in high-throughput. This platform consists of cell-based assays, imaging devices for acquisition and software solutions for the quantification of the imaging data. Importantly, aCDc saves the original and the processed images as log files, which can always be traced back to confirm appropriate quantification. aZEcs takes into account pipetting artefacts and eliminates the deficits before quantification, a feature which is not found in any other available software. We validated the assay platform using the pro-migratory growth factor HGF in a cell-based model of SHH-MB. Using aCDc, we furthermore determined for the first time the migratory response profile of two long-term, one primary and two patient-derived xenograft human MB lines to a selection of eleven growth factors and cytokines with described or suspected micro-environmental impact on MB growth and dissemination.

One limitation of 2D assays is that the invasive capability of the cells cannot be addressed adequately, even when adherent cells are embedded in 3D matrix. This is due to marked differences in cells adherent to a rigid surface compared to fully embedded cells with respect to cell signalling, motility modes and plasticity of migration^25^ (and discussed and referenced in^26^), which are critical parameters for tumour cell dissemination *in vivo* as well^27^. Hence, in addition to the zone exclusion assay, we used 3D assays to explore steady state and induced motile MB cancer cell behaviour and to identify growth factors relevant for matrix invasion and dissemination. Overall, our analyses showed a good consistency between 2D and 3D assay data for strong promoters of cell dissemination such as HGF, EGF and bFGF. However, steady-state migration was markedly higher in 2D than in 3D while differences between treatments were subtler. In contrast, and particularly striking for the spheroids, steady-state migration is restricted in 3D and requires a stimulus. This indicates that MB cells on a stiff substrate such as glass or plastic migrate spontaneously into cell free areas and suggests that the molecular mechanisms underlying motility control differ between 2D and 3D conditions in these cells.

Time-lapse movies indicated that cell movement in 3D occurs mostly outward with respect to the surface of the bead or the spheroid (videos 1–6). Mean distances between cells and beads/spheroids calculated based on the projected images using aMDIcs and aSDIcs correspond approximately to the expected effective distances, providing their position is within a 25° angle above or below the horizontal axis (Fig. S4). The quantified distance between cells and beads/spheroids outside this angle is underestimated proportional to the respective angle between cell, bead/spheroid surface and horizontal axis (Fig. S4B). However, as the depth of field is centered on the horizontal axis, the vast majority of objects analyzed by the software are within 25° off the horizontal axis and the underestimation error equal under all conditions can be considered marginal.

When aZECs is used in combination with mitogenic factors such as HGF, we recommend a time-lapsed analysis of the progression of zone exclusion, to minimize proliferation-dependent artefacts by selecting time points when proliferation effects can be considered marginal. Proliferation is less problematic in the 3D assays, as aMDIcs and aSDIcs measure distances and automatically count the number of disseminated cells. aMDIcs yielded higher variance in the distances of invasion in individual DAOY samples compared to UW228 cells. We ascribe these differences to the cell-specific capability to adhere to the cytodex microcarrier beads, which is greater for UW228 cells and appears to increase the threshold of pro-migratory cues necessary to promote detachment and dissemination. No pronounced difference in distance of migration was observed, indicating that the mode of cell growth prior to migration – spheroid versus monolayer on bead - is not determining the degree of response in responsive MB cells. However, cells in spheroids remained clustered even after treatment with bFGF, which overall triggers massive dissemination. Hence, only a subset of cells responds to growth factors with dissemination in spheroids, while the majority of cells disseminate from microbeads. What restricts spheroid cell dissemination is unknown but it is possible that cell-cell contact, which is tight in spheroids and relatively loose on microbeads, is an additional determinant for the migratory outcome. The morphodynamic processes underlying cell dissemination from spheroids or microbeads can be further addressed using lifeact-EGFP expression, which allows visualizing F-actin dynamics in disseminating cells in real time^28^ (Videos 1–6). Live cell imaging combined with confocal microscopy of MB cells migrating from microbeads (Fig. S2), indicated that dissemination relies on mesenchymal migration. This migration appears to be driven by F-actin-rich, dynamic protrusions at the leading edge of the migrating cells and supported by matrix remodelling (collagen fibre tethering (S2Aa), collagen degradation (S2Ab) and tunnel formation (S2Bc)).

Genomic analyses of large cohorts of MB now allow discriminating four molecular subgroups with defined molecular, functional and clinical characteristics^18^,^29^. We propose here a complementary method of MB subgroup characterisation that is based on the growth factor sensitivity profiles of the tumours. Although refinement will be needed and larger sample sizes necessary for standardization, this method may enable the rapid diagnostic evaluation of cells extracted from the primary tumour. Indeed, we can discriminate the established laboratory lines and the SHH PDX line Med 1712 from the non-WNT/SHH line ZH-513 and the group 3 PDX line Med 411, as latter two display selective sensitivities to NGF and PIGF-1. In addition, the high throughput capabilities of our platform in combination with primary or PDX material permit efficient co-clinical testing of potential anti-metastatic drugs. Since the assay is based on cell dissemination triggered by effective signalling through the relevant receptors and their underlying signalling pathways, it also reveals the growth factor sensitivity of a given tumour. Unlike gene expression data, this functional, co-clinical assessment of patient-derived material may provide biologically relevant information on the tumour. An example is PlGF-1 signalling, which along with its receptor neuropilin 1 is expressed in the majority of human MB and specifically contributes to growth and spread of group 3 tumours^30^. Our data in the non-WNT/SHH line ZH-513 and the group 3 PDX line Med 411 now support the notion of the specific, pro-tumorigenic impact of PlGF-1 in non-WNT/SHH MB. They furthermore indicate a pro-migratory function of PlGF-1 in MB that may explain reduced metastasis observed in orthotopic group 3 and group 4 murine models of human MB after PlGF-1 blockade^30^.

Targeting cell motility alone might not be sufficient to eradicate the cancer; however, it may contribute to its control by restricting local infiltration, by limiting further dissemination and by preventing the evolution towards a more aggressive phenotype^3^. HGF^21^,^23^,^31^,^32^ and EGF^33^ were previously shown to promote motility of MB cells. No pro-migratory functions of bFGF have been described in MB so far. In contrast, bFGF was found to interfere with SHH signalling in neuronal precursor and tumour cells^34^ and to block tumour formation in a mouse model of SHH MB^35^. Thus, although bFGF treatment elicits anti-tumorigenic responses in susceptible cell lines, its dissemination promoting functions calls for caution when bFGF treatment is considered as therapeutic strategy. HGF, EGF and bFGF activate receptor tyrosine kinases that likely feed into the same downstream signalling pathways. Therefore, an ideal target for a future drug would be a molecular hub that integrates signals from all three pathways. With MAP4K4 we recently identified such a potential convergence kinase downstream of HGF function in MB^23^, which is also activated by EGF^36^ and TNFa^37^ to promote cell motility. aCDc also identified the specific response of UW228 cells to IL-6 stimulation. Hence, in this case, an ideal target would be an effector that orchestrates HGF, EGF, bFGF and IL-6 pathways. This implicitly proves that aCDc may be used to identify specific differences among the cell types, which could be exploited to effectively target the relevant pathways.

In conclusion, we provide a novel method of cancer cell evaluation according to their growth factor-dependent dissemination behaviour. We expect this method to improve accuracy of diagnosis and ultimately aid in the tumour-specific refinement of treatment schemes for patients, to increase cure rates and to reduce treatment related morbidities.

## Methods

### Ethics Statement

Informed consent was obtained from subjects and all research involving subject’s material was conducted under appropriate review/privacy board protocols of the Kantonale Ethikkommission Zürich (Ethics Commission of the Canton of Zürich, Switzerland). The use of patient tumour material for diagnostic and prognostic analysis was approved by the Kantonale Ethikkommission Zürich.

### Reagents

Growth factors and inhibitors were used throughout the study in the concentrations indicated. Hepatocyte growth factor (HGF): 20 ng/ml (100-39), epidermal growth factor (EGF): 30 ng/ml (100-47), Insulin like Growth Factor 1 (IGF): 20 ng/ml (100-11), Netrin: 200 ng/ml (R&D Systems, 6419-N1-025), Platelet Derived Growth Factor-B (PDGF-B): 20 ng/ml (P100-14B), Tumour Necrosis Factor-α (TNF- α): 25 ng/ml (300-01A), basic Fibroblast Growth Factor (bFGF): 100 ng/ml (100-18B), Interleukin-6 (IL-6): 20 ng/ml (200-06), Nerve Growth Factor-β (NGF): 50 ng/ml (450-01), Transforming Growth Factor-β (TGF-β): 20 ng/ml (100-21) and Placental Growth Factor-1 (PlGF-1): 10 ng/ml (100-06) from PeproTech (London, UK), PHA-665752, 125 nM (Selleckchem, Houston, TX, USA, S1070), ARQ197, 125 nM (Active Biochemicals, Wanchai, Honkong, A-1109).

### Human MB cell lines, human primary MB cell and patient-derived xenograft (PDX) culture

DAOY human MB cells (originally derived from a desmoplastic cerebellar MB) were purchased from the American Type Culture Collection (ATCC, Rockville, MD, USA). UW228^38^ was generously provided by John Silber (Seattle, USA). DAOY and UW228 cells were cultured as described in^39^. DAOY LA-EGFP and UW228 LA-EGFP cells were produced by lentiviral transduction of DAOY and UW228 cells with pLenti-LA-EGFP^23^. Human primary medulloblastoma cells were derived from a resected tumour of a nine-year-old patient with an M2 stage medulloblastoma with cerebellar metastases. Histology indicated non-SHH, non-WNT type^40^. Tumour tissue was acutely dissociated using a papain-based dissociation kit (Worthington Biochemical, Lakewood, NJ) according to the manufacturer’s protocol. Following dissociation, cells were cultured in neurobasal medium (Invitrogen/Life Technologies, Paisley, UK, 12349-015) supplemented with 2% B-27® (Gibco/Life Technologies, 10889-038), 1% L-Glutamine (Invitrogen/Life Technologies, 25030024), 10 μg/ml bFGF and 10 μg/ml EGF. The human SHH PDX line Med-1712FH and the Group3 line Med-411FH were obtained from the brain tumour resource laboratory of the Fred Hutchinson Cancer Center and maintained in the same growth medium as the primary tumour cell.

### Zone infiltration assay

Oris™ 96-well cell seeding stoppers were inserted in μ-clear 96 well plate (Greiner CELLSTAR®, Frickenhausen, Germany, 655090) and 3.5 × 10^4^ cells/well were seeded. Cells were incubated at 37 °C overnight to form the zone of exclusion. The following day, after plug removal, the cells were treated with growth factors/cytokines or with HGF and PHA-665752 or ARQ197. Cell migration was monitored for 25 h using an automated ImageXpress Micro 2 microscope (Molecular Devices, LLC, USA) equipped with environmental control. Arrays of 5 × 5 images per well were acquired at 5 h intervals with a 10 × 0.2 NA Plan Apo objective (Nikon) and a Roper CoolSNAP HQ camera (Roper Scientific, Martinsried, Germany). Cell migration was measured as percentage of cell free area covered by the cells in a given time (time lapsed or endpoint) using the automated computer-assisted cell migration quantification software aZEcs.

### Microbead invasion assay

Approximately 500 Cytodex Microcarrier beads (Sigma Aldrich, St. Louis, MO, USA, C3275) per 1.25 × 10^4^ cells/ml were mixed in FACS tubes (Becton, Dickinson and Company, Allschwill, Switzerland, Falcon T7597-5J) and incubated at 37 °C for 6 h with a mild shaking of the tubes at every one hour. Cells, which were not adhered to the beads, were removed by washing the beads with fresh medium. Cell-coated microbeads were re-suspended in 2.5% bovine collagen 1 (Advanced BioMatrix, San Diego, CA, USA, 5005-B) and seeded in μ-clear 96 well plate (Greiner CELLSTAR®, 655090). Following the polymerization of collagen, the cell coated microbeads were overlaid with fresh medium and treated with appropriate concentrations of growth factors/cytokines or with HGF or with c-Met inhibitors. After 24 h, cells were fixed with 4% paraformaldehyde (PFA) and stained with Hoechst. Images were acquired using an ImageXpress Micro 2 automated microscope (Molecular Devices, LLC, USA) as described for the zone infiltration assay. Cell invasion is expressed as the average of the distance invaded by the cells from the circumference of the bead as measured by our cell dissemination counter software aMDIcs.

### Spheroid invasion assay

1000 cells/100 μl per well were seeded in a 96 well Lipidure®-Coat plate A-U96 (Amsbio, Bioggio-Lugano, Switzerland, AMS.51011610). The cells were incubated at 37 °C overnight to form spheroids. 70 μl of the medium were removed from each well, and remaining medium with spheroid was overlaid with 2.5% bovine collagen 1. Following the polymerization of collagen, fresh medium was added to the cells and treated with growth factors/cytokines or with HGF and/or c-Met inhibitors. The cells were allowed to invade the collagen matrix for 24 h, after which they were fixed with 4% PFA and stained with Hoechst. Images were acquired on an Axio Observer 2 mot plus fluorescence microscope (Zeiss, Munic, Germany) using a 5× objective. Cell invasion is determined as the average of the distance invaded by the cells from the center of the spheroid as determined by our cell dissemination counter software aSDIcs.

### Live imaging

#### Microbead invasion assay

Cytodex microcarrier beads were coated with cells according to the protocol described above. The cell-coated microbeads re-suspended in collagen were seeded in 8 well ibidi plate (ibidi, Martinsried, Germany 80821). After collagen polymerization, the cell coated microbeads were overlaid with fresh medium and treated with appropriate concentrations of growth factors/cytokines. Cell invasion was monitored for 18 h using a Axio Observer 2 mot plus fluorescence microscope (Zeiss). Images were acquired using a 20× objective at the interval of 30 minutes and the time point images were assembled into a QuickTime video (5 fps).

#### Spheroid invasion assay

500 cells/40 μl per well were seeded in a Perfecta3D® 96 well hanging drop plate (3D Biomatrix, Ann Arbor, MI, USA, HDP1096) according to the manufacture’s instructions and incubated at 37 °C for 72 h to form spheroids. The spheroids were harvested onto an 8 well ibidi plate (one spheroid per well) via the top access hole method as described by the manufacturer. The spheroids were overlaid with 2.5% bovine collagen 1. Following the polymerization of collagen, fresh medium was added to the cells and treated with growth factors/cytokines. Cell invasion monitoring and assembly of the video was performed similar to microbead invasion assay (live imaging) protocol.

### Software development

#### aZEcs

For automatic evaluation of zone exclusion, the algorithm determines the percentage of a cell-free area at time point *T*_*1*_ that remains uncovered by cells at a later time point *T*_*2*_. In the first step, the microscopy images are converted into dichroic black and white images according to a RGB threshold, where white fields correspond to areas covered by cells. This RGB threshold is calculated individually for every image based on the user-defined parameter *rgbt*, which represents the percentage of white pixels after conversion. By adjusting this parameter, the user is able to account for over- and underexposed images. Due to the high content of visible cells in the images, the default setting for the zone exclusion assay is *rgbt *= 60. In the second step, a clustering algorithm determines the size of the cell-free area in the images recorded at *T*_*1*_. Starting with a single black pixel, all adjacent black pixels are joined to form a cluster, and this procedure is recursively continued until no additional pixel can be added. Then, the next black pixel not yet belonging to a cluster is chosen and processed until a set of clusters is obtained. The cluster with the maximum area found in this procedure is defined as the cell-free area. The remaining cell-free area at time point *T*_*2*_ is expressed as the percentage of cell-free area at *T*_*1*_.

#### aMDICs

For automatic evaluation of cell motility using the microbeads dissemination/invasion assay, aMDIcs first performs bead identification. The acquired image is again converted to black and white as for the zone exclusion assay. Due to the reduced number of cells compared to the zone exclusion assay, the default value for the dichroic conversion is *rgbt *= 1. A naïve brute force algorithm then systematically scans the whole image for circular, white structures. It does so by shifting a growing circle over the image from left to right and from top to bottom. For every position and diameter, the algorithm calculates the percentage of the circle that is covered by white pixels in the dichroic image. All objects that cover more than a user-defined fraction (default = 50%) of the circle with white pixels are considered as a bead. For these objects, the algorithm stores the centre of the circle and the respective diameter. After scanning the image, the algorithm recursively selects the beads with the highest coverage and extracts them from the original image for analysis. This is done to ensure that the respective local optimum (in terms of bead centre and diameter) is chosen for every bead. Then the bead-to-bead distances are calculated and only isolated beads without potentially overlapping migration areas are kept. Cells are identified using the clustering procedure described in the aZEcs algorithm. For aMDIcs and aSDIcs, the neighbourhood definition during the clustering process is user-defined and may allow black pixels in between white clusters in order to detect scattered cells as single clusters. A user-defined minimal and maximal cluster size corresponding to the average area of a single nucleus is applied to identify individual cells. All clusters smaller than the minimum value are considered as debris and removed. Clustered pixels covering an area larger than the defined maximum value are considered as clustered cells. The area of such a cluster is divided by the average area of all nuclei analysed, yielding the approximate number of cells contributing to that cluster. All clusters containing white pixels adjacent to the bead surface are removed, as these pixels derive from cells that have not migrate away from the bead. For the remaining clusters, the mean distance between all pixels and the bead centre is calculated. A histogram depicting the cumulated means is generated (Fig. 2) and a log-file, containing the means and standard deviations is produced. For aMDIcs, an additional histogram with the combined data of all located beads is generated to depict variations between beads.

#### aSDIcs

For automatic evaluation of cell motility using the spheroid dissemination/invasion assay, the images are also converted into a dichroic image. White pixels in close proximity are then clustered and processed as described for aMDIcs. In contrast to the microbeads dissemination/invasion assay, in the spheroid invasion assay no bead surface is available as reference point. The spheroid is therefore converted into a single cluster of white pixels and the centre of this cluster defines the reference point for distance quantification.

### Statistical analysis

Mean ± SD are shown when means of three independent experiments are compared, and box plots with whiskers to min and max are shown when multiple individual measurements from three independent experiments (except Fig. 5B) are compared. Unpaired student’s t-test was used to test significance of differences between two samples in zone infiltration experiments (Fig. 3B–E and S1). For all other analyses one-way ANOVA repeated measures test using Bonferroni’s Multiple Comparison was performed. *P*-Values < 0.05 were considered significant. (*p ≤ 0.05, **p ≤ 0.01, ***p ≤ 0.0001). Where indicated, asterisks show statistical significances between control and test sample.

## Additional Information

**How to cite this article**: Kumar, K. S. *et al.* Computer-assisted quantification of motile and invasive capabilities of cancer cells. *Sci. Rep.* **5**, 15338; doi: 10.1038/srep15338 (2015).

## Supplementary Material

Supplementary Information

Supplementary Information

Supplementary Information

Supplementary Information

Supplementary Information

Supplementary Information

Supplementary Information
