# Corrigendum: Computer-assisted quantification of motile and invasive capabilities of cancer cells

**DOI:** 10.1038/srep46996

**Published:** 2018-07-02

**Authors:** Karthiga Santhana Kumar, Max Pillong, Jens Kunze, Isabel Burghardt, Michael Weller, Michael A. Grotzer, Gisbert Schneider, Martin Baumgartner

Scientific Reports 5; Article number: 1533810.1038/srep15338; published online: 10 21 2015; updated: 07 02 2018

In the original version of this Article, the author Karthiga Santhana Kumar was incorrectly indexed.

In addition, this Article contained an error in the Results section, under the subheading ‘Automated quantification of cell dissemination’,

“A program suite referred to as automated Cell Dissemination counter (aCDc) consisting of three open source, executable Java programs (.jar) files can be downloaded through the following web link http://www.infozentrum.ethz.ch/uploads/user_upload/Software/.”

now reads:

“A program suite referred to as automated Cell Dissemination counter (aCDc) consisting of three open source, executable Java programs (.jar) files can be downloaded through the following web link https://urldefense.proofpoint.com/v2/url?u=http-3A__www.infozentrum.ethz.ch_uploads_user-5Fupload_Software_ImageAnalysisSoftware.zip&d=DwIGaQ&c=vh6FgFnduejNhPPD0fl_yRaSfZy8CWbWnIf4XJhSqx8&r=Q0HWMVGq_tlDBf7wAxJCv189FbTXBj2Fzg4N_lVZ7NM&m=y4-cT7GLvN-bRkjsbfl2iBNdNwRNEYQ4YmdXP3tZjas&s=o6yob3XKrSnFjEfuAxXQN7WgIuJhyqBCi8hBAm94hFg&e=.”

