# Supplementary material for: Computer-assisted quantification of motile and invasive capabilities of cancer cells
Source: Sci Rep. 2015 Oct 21;5:15338. doi: 10.1038/srep15338 (PMC4614254; doi:10.1038/srep15338)
Supplement: Supplementary Information [file srep15338-s1.pdf]

Computer-assisted quantification of motile and invasive capabilities of cancer cells

Karthiga Santhana Kumar[1], Max Pillong[2,5], Jens Kunze[2,5], Isabel Burghardt[3], Michael Weller[3], Michael A. Grotzer[1,4], Gisbert Schneider[2] and Martin Baumgartner[1,6]

Supplementary information

[1]: Department of Oncology, Children's Research Center, University Children's Hospital Zürich, August-Forel Strasse 1, CH-8008 Zürich, Switzerland

[2]: Department of Chemistry and Applied Biosciences, ETH Zürich, Vladimir-Prelog-Weg 4, CH-8093 Zürich, Switzerland

[3]: Department of Neurology, University Hospital Zürich and University of Zürich, Frauenklinikstrasse 26, CH-8091 Zürich, Switzerland

[4]: Department of Oncology, University Children's Hospital Zürich, Steinwiesstrasse 75, CH-8032 Zürich, Switzerland

[5]: These authors contributed equally to this work

[6]: Correspondence should be addressed to Martin Baumgartner (Martin.Baumgartner@kispi.uzh.ch)

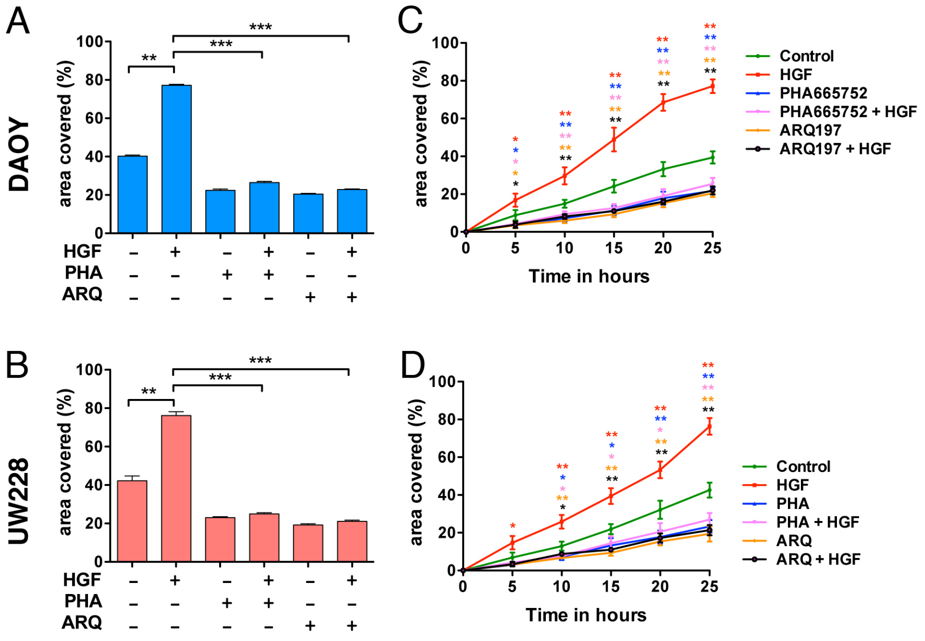

Fig. S1 aZEcs, aMDIcs and aSDIcs quantifications reliably confirm manual measurements of HGF-induced cell dissemination under serum-free conditions. A, B) Means and SDs of % area covered from three independent zone infiltration experiments using aZEcs in DAOY (A) or UW228 (B) at T_{24h} after stimulation with 20 ng/ml HGF or treatment with c-Met inhibitors PHA665752 or ARQ197 (125 nM each) or both. **C, D)** Time-lapsed mean and SD quantifications of % area covered from three independent zone infiltration experiments using aZEcs in DAOY (C) or UW228 (D) after stimulation with 20 ng/ml HGF or treatment with c-Met inhibitors PHA665752 and ARQ197 (125 nM each) or both.

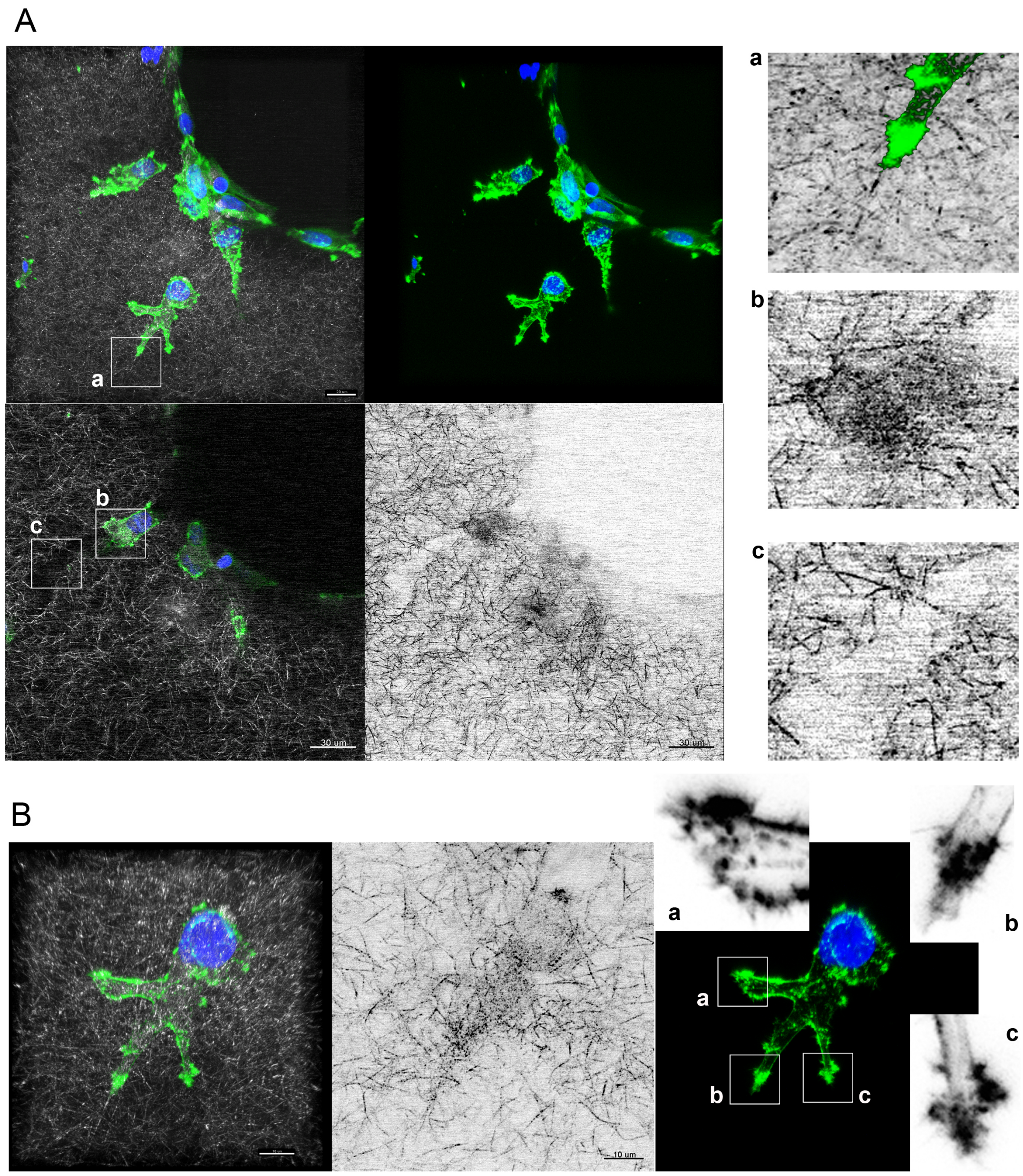

Fig. S2 Dissemination of DAOY cells from microbeads into collagen. 3D surpass images of LA-EGFP-expressing DAOY cells migrating of microbeads into collagen gel. a, b and c are 4x magnifications of the boxed areas and show invasion-dependent modifications in the collagen gel. Green: LA-EGFP, blue: hoechst DNA staining, black and white: reflection image of collagen fibres. B) Higher magnification image of single invading cell.

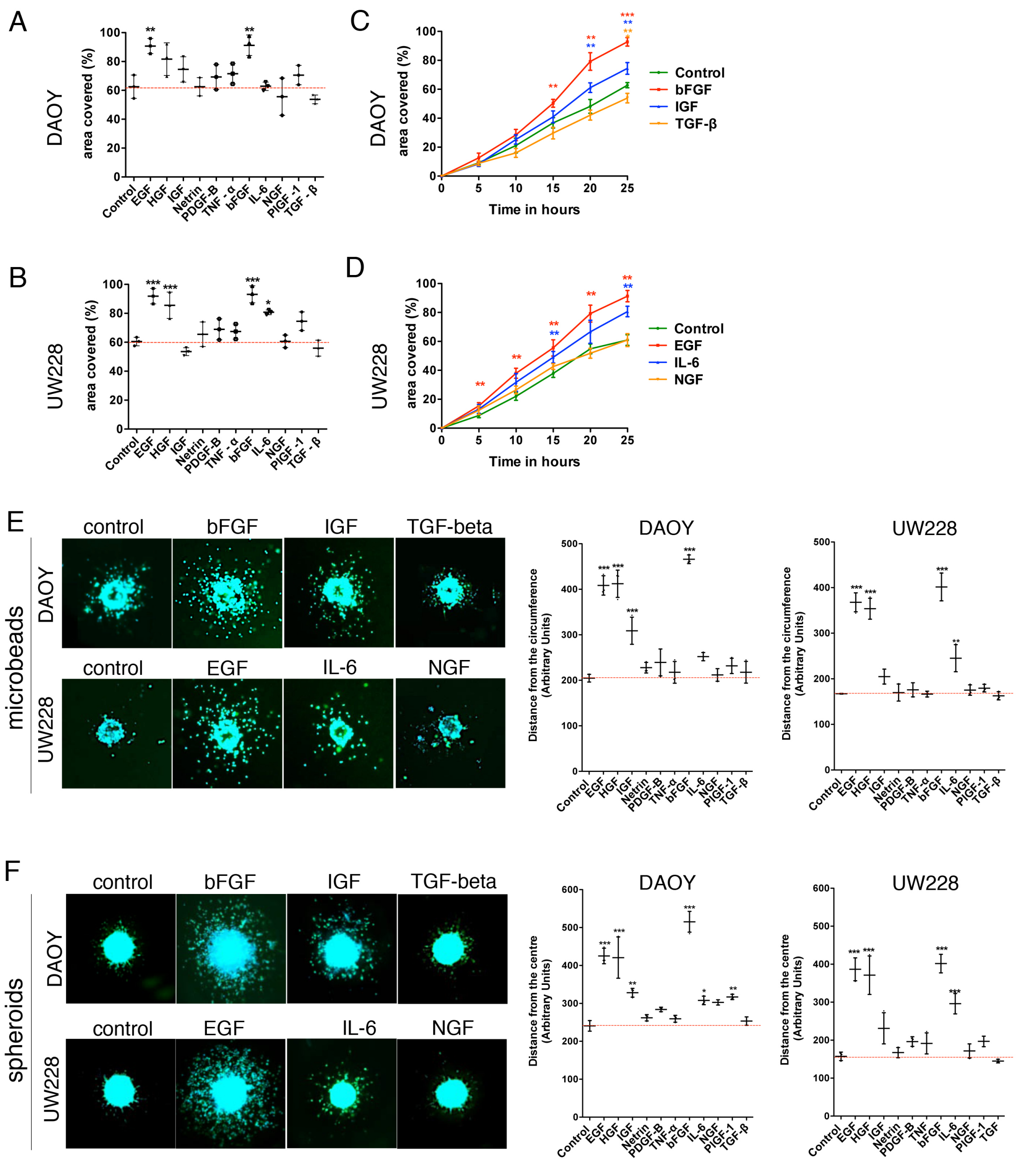

Fig. S3 Selective induction of cell dissemination with growth factors under serum-free conditions. A, B) End-point quantification of % area covered in zone infiltration assay using aZEcs. Means and SDs of three independent experiments with DAOY (A) or UW228 (B) cells at T_{24h} after stimulation with factors as indicated are shown. Concentrations of growth factors/cytokines as in 4A,B. **C, D)** Time-lapsed mean and SD quantifications of % area covered from three independent zone infiltration experiments using aZEcs in DAOY (C) or UW228 (D) cells stimulated with factors as indicated. **E)** Representative images of 100x magnified microbeads coated with DAOY or UW228 cells after 24 h -/+ stimulation with bFGF, IGF or TGF-beta (DAOY) or EGF, IL-6 or TGF-beta (UW228). LA-EGFP in green, Hoechst staining in blue. Right panels: quantification of means and SDs of cell dissemination/invasion from three independent experiments using aMDIcs. **F)** Representative images of 50x

magnified spheroids of DAOY or UW228 cells after 24 h -/+ stimulation with bFGF, IGF or TGF-beta (DAOY) or EGF, IL-6 or NGF (UW228); LA-EGFP in green, Hoechst staining in blue. Right panels: Quantification of distance of cell dissemination/invasion using aSDIcs.

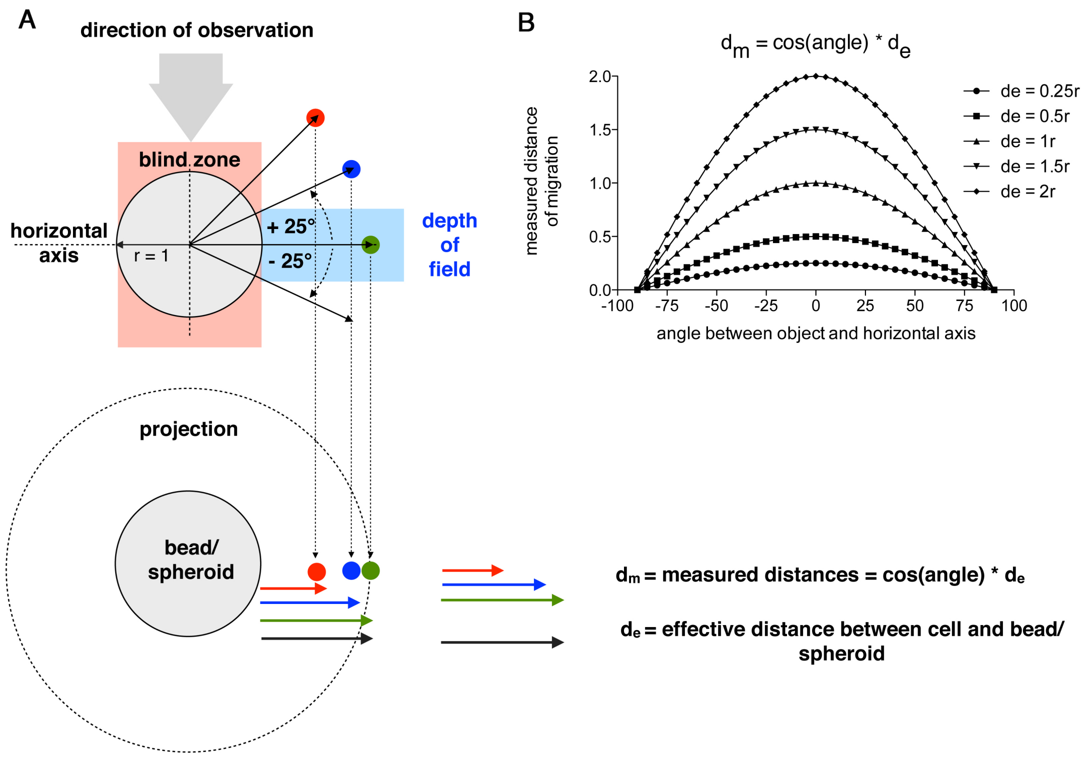

Fig. S4 Schematic overview of angle-distance relationship for distance quantification. **(A)** The images used for the aMDIcs and aSDIcs quantification are projections of 3D spaces acquired by non-confocal microscopy with a depth of field of approximately 50 μm (blue highlight). The area above and below the beads is not visible (red highlight). The projection of objects (cells) from the 3D space onto a 2D images causes the underestimation of distances according to the formula $d_m = \cos(\text{angle}) * d_e$ (d_m: measured distance, d_e: effective distance of cell from bead surface). **(B)** XY plot shows relationship between angle of observation and the deviation of the measured distance from the effective distance. Measured distances of objects located within 25° above and below the horizontal axis deviate from the effective distance between object and beads surface by less than 10%.

Supplementary Video 1

Collagen embedded microbead coated with DAOY cells. Inverted grey scale of EGFP fluorescence of DAOY cells expressing LA-EGFP. Cells were seeded on microbeads and embedded in collagen. 20 x objective, recording time 18 h, 30 min intervals, 10 frames/second.

Supplementary Video 2

Collagen embedded microbead coated with DAOY cells and stimulated with HGF. Inverted grey scale of EGFP fluorescence of DAOY cells expressing LA-EGFP. Cells were seeded on microbeads. embedded in collagen and stimulated with 20 ng/ml HGF. 20 x objective, recording time 18 h, 30 min intervals, 10 frames/second.

Supplementary Video 3

Collagen embedded microbead coated with UW228 cells. Inverted grey scale of EGFP fluorescence of UW228 cells expressing LA-EGFP. Cells were seeded on microbeads and embedded in collagen. 20 x objective, recording time 18 h, 30 min intervals, 10 frames/second.

Supplementary Video 4

Collagen embedded microbead coated with UW228 cells and stimulated with HGF. Inverted grey scale of EGFP fluorescence of UW228 cells expressing LA-EGFP. Cells were seeded on microbeads and embedded in collagen and stimulated with 20 ng/ml HGF. 20 x objective, recording time 18 h, 30 min intervals, 10 frames/second.

Supplementary Video 5

Collagen embedded DAOY spheroid: Inverted grey scale of EGFP fluorescence of DAOY cells expressing LA-EGFP. Cells were grown to spheroids and embedded in collagen. 20 x objective, recording time 18 h, 30 min intervals, 10 frames/second.

Supplementary Video 6

Collagen embedded DAOY spheroid stimulated with EGF: Inverted grey scale of EGFP fluorescence of DAOY cells expressing LA-EGFP. Cells were grown to spheroids, embedded in collagen and stimulated with 30 ng/ml EGF. 20 x objective, recording time 18 h, 30 min intervals, 10 frames/second.
